# Structure and oligomerization of the periplasmic domain of GspL from the type II secretion system of *Pseudomonas aeruginosa*

**DOI:** 10.1038/s41598-018-34956-w

**Published:** 2018-11-13

**Authors:** Aleksandra Fulara, Isabel Vandenberghe, Randy J. Read, Bart Devreese, Savvas N. Savvides

**Affiliations:** 1 Unit for Structural Biology, Department of Biochemistry and Microbiology, Ghent University, 9052 Ghent (Zwijnaarde), Belgium; 2 VIB-UGent Center for Inflammation Research, 9052 Ghent (Zwijnaarde), Belgium; 3 Laboratory for Microbiology, Department of Biochemistry and Microbiology, Ghent University, 9000 Ghent, Belgium; 4 Department of Haematology, Cambridge Institute for Medical Research, University of Cambridge, Cambridge, CB2 0XY United Kingdom

## Abstract

The ability of bacteria to infect a host relies in part on the secretion of molecular virulence factors across the cell envelope. *Pseudomonas aeruginosa*, a ubiquitous environmental bacterium causing opportunistic infections in humans, employs the type II secretion system (T2SS) to transport effector proteins across its cellular envelope as part of a diverse array of virulence strategies. General secretory pathway protein L (GspL) is an essential inner-membrane component of the T2SS apparatus, and is thought to facilitate transduction of the energy from ATP hydrolysis in the cytoplasm to the periplasmic components of the system. However, our incomplete understanding of the assembly principles of the T2SS machinery prevents the mechanistic deconvolution of T2SS-mediated protein secretion. Here we show via two crystal structures that the periplasmic ferredoxin-like domain of GspL (GspL^fld^) is a dimer stabilized by hydrophobic interactions, and that this interface may allow significant interdomain plasticity. The general dimerization mode of GspL^fld^ is shared with GspL from *Vibrio parahaemolyticus* suggesting a conserved oligomerization mode across the GspL family. Furthermore, we identified a tetrameric form of the complete periplasmic segment of GspL (GspL^peri^) which indicates that GspL may be able to adopt multiple oligomeric states as part of its dynamic role in the T2SS apparatus.

## Introduction

Over millions of years of coexistence pathogenic bacteria have developed diverse molecular strategies to achieve colonization and infection of their hosts. In order to reach host tissues a bacterial toxin or lytic enzyme has to first cross the bacterial cell envelope. To facilitate transport of the virulence factors and to enable communication with their external environment Gram-negative bacteria utilize at least six conventional multi-protein nanomachineries called secretion systems^1,2^.

*Pseudomonas aeruginosa* is an opportunistic pathogenic bacterium which infects immune compromised humans such as cystic fibrosis patients. These infections are characterized by high morbidity and mortality^3^. Due to abundance of multi-drug resistant *P*. *aeruginosa* strains in hospital settings, in 2017 WHO has identified the bacterium as of critical importance for which there is an urgent need for new treatments^4^. Five out of six secretion systems are present in the *P*. *aeruginosa* arsenal^5^, and its type II secretion system (T2SS) is responsible for the transport of the widest variety of molecular cargos^6^. Structurally the T2SS is a nanomachinery spanning the bacterial inner- and outer-membrane, which by the virtue of multiple protein-protein interactions orchestrates the translocation of folded proteins from the periplasm to the external environment^7^. Due to the fact that the T2SS is commonly employed by Gram-negative bacteria this system has also been termed the main terminal branch of the **g**eneral **s**ecretory **p**athway, and its constituent proteins as Gsp proteins.

In the case of *P*. *aeruginosa*, the T2SS is composed of 12 oligomeric proteins representing four functional subassemblies: the T2SS ATPase GspE residing in the cytoplasm; GspF, L, M and C composing the inner membrane platform; the pseudopilus formed by GspG, H, I, J and K which is anchored in the inner membrane but extending to the periplasm; and the outer-membrane secretin GspD. The twelfth Gsp protein, GspO, is only transiently associated with the machinery to carry out enzymatic cleavage of pseudopilus proteins necessary for the assembly of the appendage^5^.

The inner-membrane bitopic protein GspL can be considered a central player in the T2SS because it appears to interact extensively with other components of the system. In the cytoplasm it associates with GspE and GspF^8–10^, and in the inner membrane and/or in the periplasm it interacts with GspM^11–13^. In an overexpression scenario, the latter interaction protects GspL from proteolytic degradation *in vivo*^11,12^. GspL has also been crosslinked with GspG *in vivo*, which suggested the transient interaction between the inner membrane platform and the pseudopilus^14^. Even though the exact mode of secretion via T2SS has not been fully elucidated, the role of GspL is anticipated to help in linking adenosine triphosphate (ATP) hydrolysis to the assembly of the pseudopilus, which together constitute the two crucial steps in exoprotein transport^7,15,16^. The recent cryo-electron tomography studies on the type IV pilus system (T4PS), a nanomachinery important for bacterial motility which shares evolutionary origins with the T2SS provided the first holistic model of the T4PS^17^. In this model the GspL-GspM homologous complex is assumed to play a role in passing the signal for pilus retraction, consistent with previous works^17–19^. In this regard, involvement of GspL in these essential yet poorly understood periplasmically localized stages of T2SS-dependent secretion calls for a structural and mechanistic dissection of its oligomeric propensities.

Intriguingly, such properties have not been reported in solution, and the available data on the oligomeric state of soluble domains of GspL were derived either from crystal structures^20,21^, and/or the stoichiometry of the complex formed with an interaction partner^15,22^. While such information has served to fuel the construction of T2SS models^7,15^, it likely only provides a partial view of the actual T2SS assembly. Interestingly, the recent structural studies by cryo-electron microscopy of bacterial secretins have revealed assemblies obeying 14–15- and 16-fold symmetry^23–25^. Such new observations have challenged the paradigmatic dodecameric assembly of T2SS secretins^26,27^, and illustrate the importance of pursuing structural studies across a broad spectrum of homologous T2SS components. Moreover, the possibility that at least some of the Gsp proteins might exist in multiple oligomeric states would be in line with the recently recognized dynamic character of the T2SS^2,26,28–30^.

Appreciating the necessity to accommodate oligomeric form of an individual component when arranging the functional secretion system, we here provide structural insights into the oligomeric propensities of the periplasmic component of GspL in solution. Moreover, by employing two distinct crystal structures we provide support for the biological relevance of the dimeric hydrophobic interface of GspL^fld^^21^ and present evidence on the possible structural plasticity of the dimer. Collectively, our study sheds light onto the T2SS mode of action and provides new data to fuel a consensus about the functional stoichiometry of the system.

## Results

### Recombinant GspL^peri^ adopts distinct oligomeric forms

**Figure 1:**
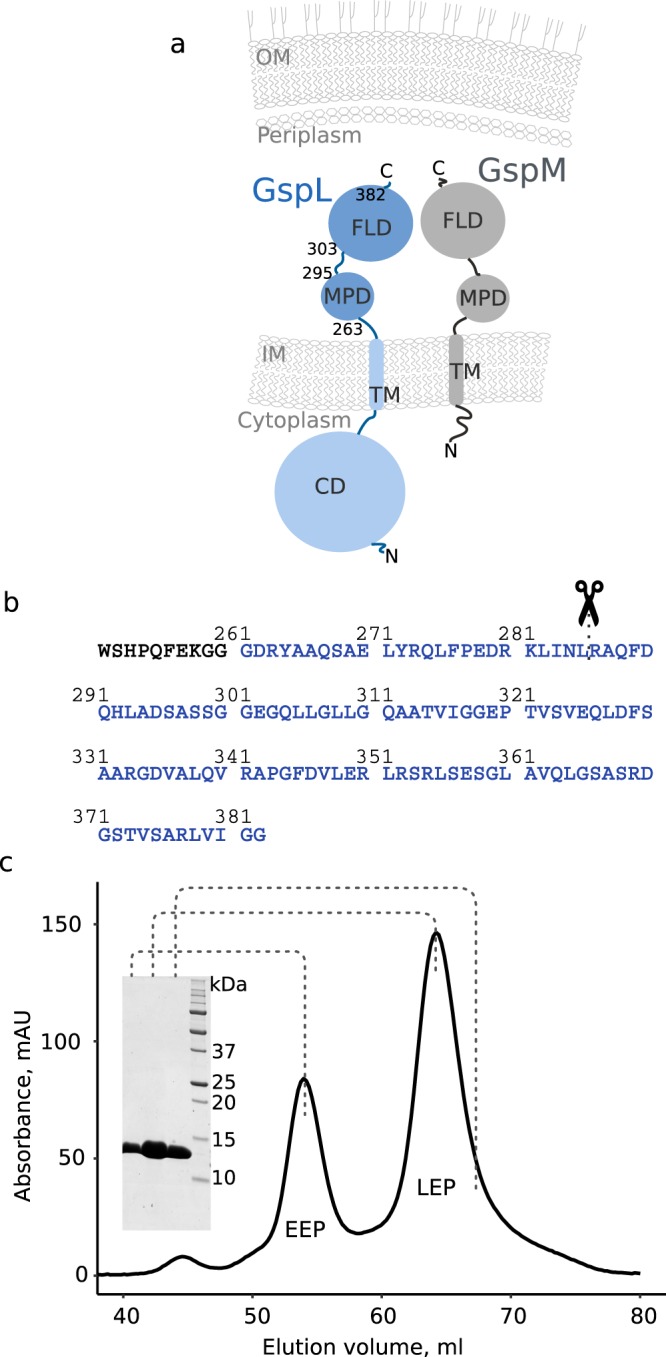
Domain organization, construct design and purification of GspL. (**a**) Schematic representation of GspL (blue) and its positioning in the bacterial inner-membrane (IM). FLD: ferredoxin-like domain, MPD: membrane proximal domain, CD: cytoplasmic domain. Numbering corresponds to the domain boarders, which can be traced to the sequence in panel b. Except for the cytoplasmic part, GspL shares the domain organisation with GspM (grey), its well established interaction partner. (**b**) Protein sequence of the construct used in the study. GspL sequence is in blue, whereas purification tag sequence is in black. Serendipitously, in the course of crystallization experiment, GspL^peri^ was proteolytically cleaved in the middle of MPD (indicated with scissors), which lead to crystallization of the shorter construct, herein termed GspL^fld^ to distinguish it from the initially purified recombinant GspL^peri^ (**c**) SEC profile of GspL^peri^ accompanied by SDS-PAGE analysis of the indicated peak fractions. EEP: early elution peak, LEP: late elution peak.

In the context of the T2SS inner-membrane platform, GspL is a bitopic protein possessing cytoplasmic and periplasmic domains (Fig. 1a). In our studies we have focused on the periplasmic segment of GspL (historically called XcpY) from *P*. *aeruginosa*, GspL^peri^, which consists of two domains: a membrane proximal domain (MPD) connected via a short linker to a ferredoxin-like domain (FLD; Fig. 1a,b). Besides the cytoplasmic part, GspL shares the domain organization with GspM, its inner membrane platform interaction partner (Fig. 1a).

Expression tests revealed that only a construct with an N-terminal Strep-tag could be produced to satisfactory amounts, in contrast to C- and N-terminally His-tagged constructs (Fig. 1b,c). During size-exclusion chromatography (SEC), the 14 kDa GspL^peri^ eluted as two peaks and electrophoresed at the same height on sodium dodecyl sulphate polyacrylamide gel electrophoresis (SDS-PAGE) (Fig. 1c), suggesting the co-existence of two oligomeric forms.

### Irreversible tetramer to dimer transition of GspL^peri^ in solution

To further investigate the apparent existence of two distinct oligomeric states of GspL^peri^, purified GspL^peri^ was subjected to SEC coupled to in-line multi-angle laser light scattering (SEC-MALLS) analysis, which allows reliable estimation of the molecular mass of protein oligomeric states and stoichiometries under native conditions^31,32^. We note that reported estimates of the oligomeric state of the homologous protein GspL from the T2SS of *Vibrio parahaemolyticus* only relied on SEC analysis and dynamic light scattering, two methods that depend exclusively on the hydrodynamic radius of the molecular species under study^21^.

**Figure 2:**
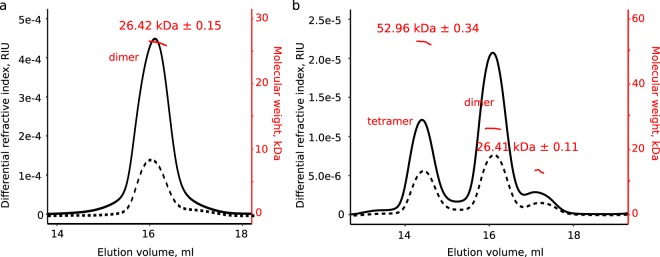
GspL^peri^ adopts dimeric and tetrameric forms as revealed by SEC-MALLS (**a**) SEC-MALLS analysis of late elution peak of GspL^peri^ shows a dimeric form (dashed line), and the oligomeric state does not change upon concentration (solid line). (**b**) GspL^peri^ from early elution peak diversifies into two populations, of tetramers and dimers, respectively (dashed line). Concentration of the EEP GspL^peri^ does not enrich the tetrameric population (solid line). However, the procedure results in longer time separation between the purification and the light scattering experiment, which shifts the tetramer/dimer ratio in favor of dimer. Tetramer to dimer ratio calculated as the ratio of areas under the relevant peaks is 0.70 at lower and 0.57 at higher concentration. Molecular weight of GspL^peri^ calculated from the sequence is 13.95 kDa.

As revealed in Fig. 2a, the late elution peak (LEP) corresponds to a dimeric species of GspL^peri^. Interestingly, the material from the early elution peak (EEP) underwent significant changes within the one hour elapsed between purification and SEC-MALLS runs, such that an initial homogeneous population converted into three subpopulations (Fig. 2b). We were able to assign the two most prominent fractions to tetrameric and dimeric GspL^peri^, respectively. In this regard, the SEC-MALLS profile reveals that tetrameric GspL^peri^ observed as a single peak during the purification step, converts into dimeric GspL^peri^ (Fig. 2b). In order to test if the tetramer to dimer transition is reversible and concentration dependent, the initially collected SEC fraction from the LEP was concentrated to 20 mg/ml and subjected to SEC-MALLS. Even at such a high concentration tetrameric GspL^peri^ did not form, providing evidence for the superior stability of the dimer (Fig. 2a, solid line). To investigate the transition even further, we assumed that the presence of tetramers in the system might be a prerequisite for the transition to happen. For this reason, the initial fraction from the EEP was concentrated and analyzed by SEC-MALLS (Fig. 2b, solid line). The relative population of GspL^peri^ dimers compared to tetrameric GspL^peri^ is higher as compared to the lower concentration sample (Fig. 2b, solid vs dashed line, calculated as a ratio of areas under the relevant peaks) implying that the conversion is irreversible and not concentration dependent. The molecular weight determination of the species constituting the latest elution peak in Fig. 2b is less accurate and therefore not reliable due to a much too low concentration. However, we note that the relative intensity of this peak, in reference to the sum of the dimer and the tetramer intensities, does not change in time suggesting that this species does not play a role in the observed oligomeric transitions of GspL^peri^.

Our experimental observation of two distinct oligomeric forms of GspL^peri^ provides evidence for the possible intrinsic ability of GspL to assemble into higher order oligomers. Such propensity of GspL has been suggested earlier based on the crystal structures of the cytoplasmic domain of GspL with ATPase GspE^22,29^, but has never been established in solution. Additionally, the short lifetime of the tetramer implies superior stability of the GspL^peri^ dimer in solution.

### Dimeric assembly of GspL^fld^ revealed by two distinct crystal forms

In an effort to better understand the oligomeric tendencies of GspL^peri^ we pursued extensive crystallization trials and managed to crystallize it in two distinct crystal forms (Table 1). Unexpectedly, the obtained crystals were found to contain a proteolytic product of the initial sample, as revealed by SDS-PAGE and silver staining of bands from dissolved crystals, and further confirmed by Edman degradation (Fig. S1). The newly formed N-terminus was determined to be residue 286, which corresponds to the middle of the MPD (Fig. 1a,b). However, interpretable electron density only starts at residues 304 and 303, for crystal forms 2 and 1, respectively. As a consequence, only the structure of the ferredoxin-like domain of GspL has been determined and therefore the crystallized construct will hereafter be referred to as GspL^fld^, to distinguish it from the longer GspL^peri^. Importantly, GspL^fld^ encompasses most of GspL^peri^ and hence the models can be of use for deriving the oligomeric propensities of the latter one.

**Table 1:** Crystallographic data collection and refinement statistics.

	Gsp^fld^	
	Crystal form 1	Crystal form 2
**Data collection**		
Beamline	Proxima 2	Proxima 2
Wavelength, Å	0,9801	0,9801
Space group	I 4_1_22	P 2_1_2_1_2_1_
Unit cell, Å	a = b = 100.44, c = 98.85	a = 23.40, b = 64.79, c = 98.95
Mosaicity, °	0.097	0.191
Rotation per image, °	0,1	1
Total rotation angle, °	180	105
Data anisotropy limits, Å	a = b = 2.5, c = 3.0	—
Resolution, Å	50–2.5 (2.65–2.5)	40–2.0 (2.2–2.0)
Measured reflections	117278 (17056)	43482 (7012)
Unique reflections	9039 (1419)	10733 (1707)
Completeness, %	99.7 (98.5)	99.3 (99.0)
Multiplicity	12.97 (12.02)	4.05 (4.11)
I/σ(I)	16.56 (1.36)	7.42 (1.56)
R_meas_, %	13.0 (225.6)	15.7 (93.6)
CC^1/2^, %	99.9 (54.7)	99.6 (79.2)
**Refinement**		
Reflections in refinement	9025	10720
Reflections in R_free_ set	902	1073
R_work_, %	0.201	0.222
R_free_, %	0.220	0.269
Non-H atoms	623	1270
Protein	605	1186
Ligands	6	—
Water	12	96
RMS bonds, Å	0.004	0.004
RMS angles, °	0.805	0.681
Ramachandran outliers/favoured (%)	0/98.7	0/100
Rotamer outliers/favoured (%)	0/87.1	1.65/88.62
PDB code	5n7l	6ghu

**Figure 3:**
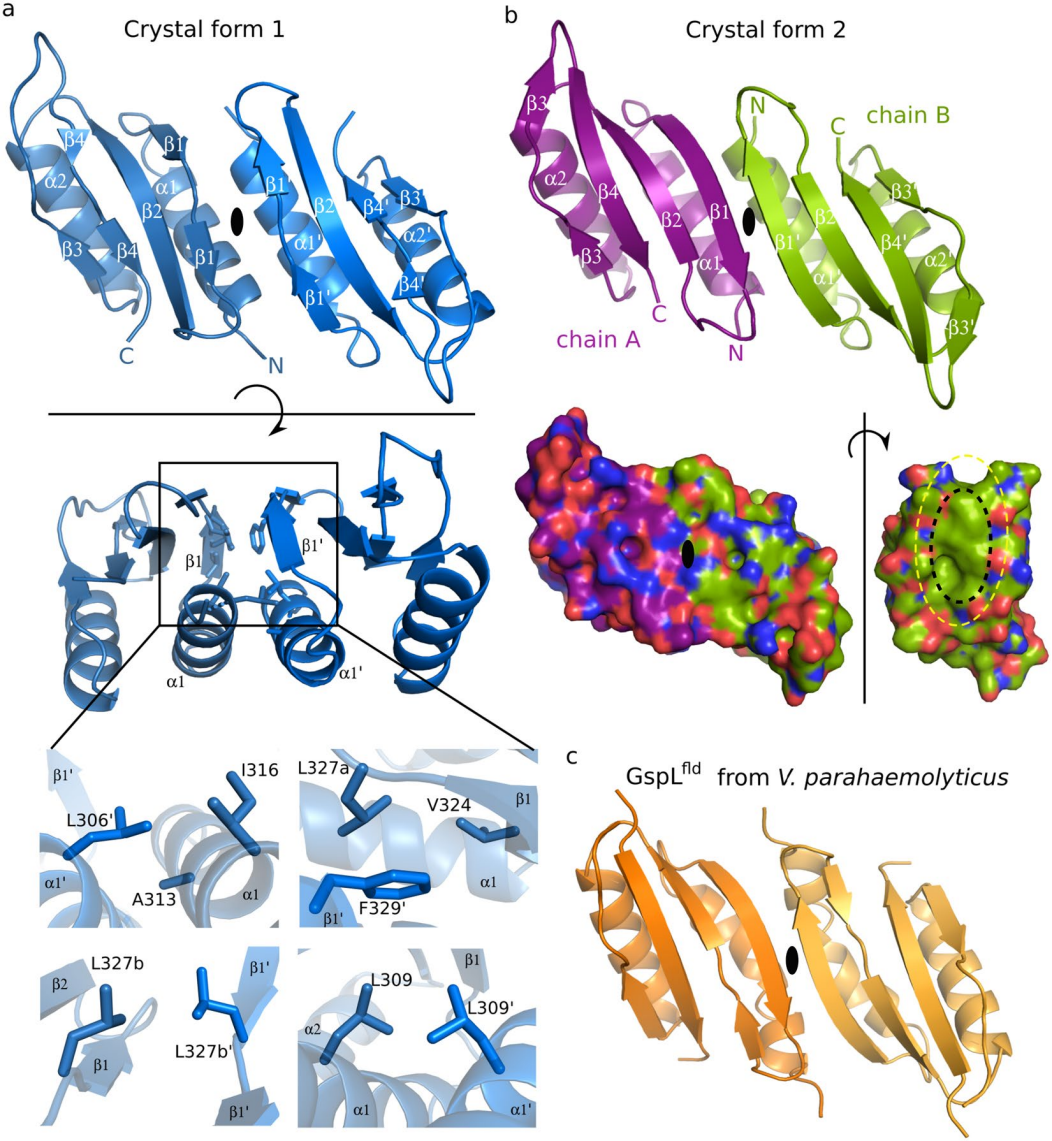
The architecture of the GspL^fld^ dimer and the hydrophobic nature of the interaction interface is conserved. (**a**) Crystal form 1 of GspL^fld^ dimer seen from the side and from the top. The inset reveals the details of the hydrophobic interface. The interacting residues are located on strand β1 and helix α1 and their symmetry related interaction partners. Leu327 is built in the model in two different conformations, labeled a and b in the bottom panel. (**b**) Side-view of crystal form 2 of the GspL^fld^ dimer. In contrast to crystal form 1, the dimer is present in the asymmetric unit but the interface also involves residues from strand β1 and helix α1 of chain A and strand β1′ and helix α1′ of chain B. In bottom panel, crystal form 2 is shown in surface representation. All atoms are colored according to the type (oxygens: red, nitrogens: blue) and carbon atoms are in the respective chain color. Display of chain B as it would face chain A at the GspL^fld^ dimer interface reveals a buried hydrophobic patch in the GspL^fld^ dimer (inner dotted ellipse). (**c**) GspL^fld^ dimer from *Vibrio parahaemolyticus*, PDB: 2w7v^21^. For details on the conservation of the interacting residues see Figure S2.

Both crystal forms unveil the dimeric assembly of GspL^fld^ (Fig. 3a,b), which in crystal form 1 is generated by a crystallographic 2-fold axis of symmetry and in crystal form 2 exists as a full dimer in the crystal asymmetric unit. The architecture of the two dimers is similar, with side chains from helices α1 and α1′ and strands β1 and β1′ contributing to the dimer interface. These features are also shared with GspL^fld^ from the T2SS of *Vibrio parahaemolyticus*^21^, shown in Fig. 3c. The continuity and length of all four strands in GspL^fld^ models is perturbed. The continuity of the strand β1 of crystal form 1 is compromised due to the adoption by Leu327 of two distinct conformations, with only one of them providing extension of the strand (bottom panel of Fig. 3a). Moreover, in our two *P*. *aeruginosa* GspL^fld^ crystal forms, and in contrast to the crystal form of GspL^fld^ from *V*. *parahaemolyticus*, helix α1 is a classical α-helix with no kink.

In contrast to the dimer of GspL^fld^ in crystal form 1 and the dimer of GspL^fld^ from *V*. *parahaemolyticus*^21^, crystal form 2 displays a full dimer in the crystal asymmetric unit. The ferredoxin-like domain of GspL adopts a rare permutation of the ferredoxin fold (βαβ)_2_, whereby the α-helix is followed by two β-strands (αββ)_2_ (Supl. Fig. 2c). Interestingly, the closest structural homologue of GspL^fld^ displaying the same infrequent permutation is a periplasmic domain of GspM^33^, which is a well-established interaction partner of GspL in the inner-membrane platform (Fig. 1a). The type II secretion machinery is evolutionary related to another bacterial multiprotein system, the type IV pilus system (T4PS)^7^, which is crucially involved in bacterial motility. Similar to the ferredoxin-like domains of GspL and GspM, also their homologues from the T4PS, PilN and PilO, possess C-terminal ferrodoxin-like domains^18,34^. Importantly, all of the above-mentioned proteins crystallized as dimers, but the dimeric interface is different in each case^18,21,33,34^.

### The dimer interface of GspL^fld^ is predominantly hydrophobic

The core of the dimer interaction interface of crystal form 1 of GspL^fld^ is almost purely hydrophobic (Fig. 3a,b). The contributing residues belong to the first half of the ferredoxin fold, namely to helix α1 and strand β1. On the α1 side, close to the N-terminus of the helix, the side chains of Leu306 and Ala313′ establish a heterotypic interaction which is complemented by Ile316′. On the opposite side of the interface another hydrophobic clamp is formed by Phe329, Leu327a′ and Val324′, the residues belonging to strand β1. The core of the interface is constituted by two interactions between symmetry related residues: the first involving Leu309 and the second involving b-conformers of Leu327 (Fig. 3a, bottom panel seen clockwise from top left).

The hydrophobic character of the interacting residues is well conserved among GspL proteins, although not a single side chain is identical among all group members (Figure S2a). The conservation is particularly strong in the case of residues at the core of the interface, namely Leu306, Leu309, Leu327 and Phe329 with at least one of the latter two having aromatic character. Interestingly, in the *V*. *parahaemolyticus* GspL^fld^ dimer both residues are aromatic and are in close proximity to each other. However, they do not interact via π-electron stacking but are involved in van der Waals interactions, more with other interface residues than among each other.

Taking into consideration participating residues, the interaction interfaces of GspL^fld^ from *P*. *aeruginosa* and from *V*. *parahaemolyticus* are qualitatively very similar. However, the buried surface area of the dimer interface in crystal form 1 amounts to 974 A^2^, which can be classified as being rather limited based on a recent survey of biologically relevant interaction interfaces^35^. In such a scenario a new crystal form may serve as a powerful tool to discriminate between biologically relevant interfaces and crystal contacts. Indeed, the two distinct crystal forms of GspL^fld^ we present here display the same type of dimer interface, which together with the structure of the homologous GspL from *V*. *parahaemolyticus* and structure-sequence considerations illustrates the conservation of the hydrophobic dimer interface among periplasmic domains of GspL proteins (Fig. 3, and Sup. Fig. S2a,b).

### Dimeric GspL^fld^ displays inter-subunit plasticity

**Figure 4:**
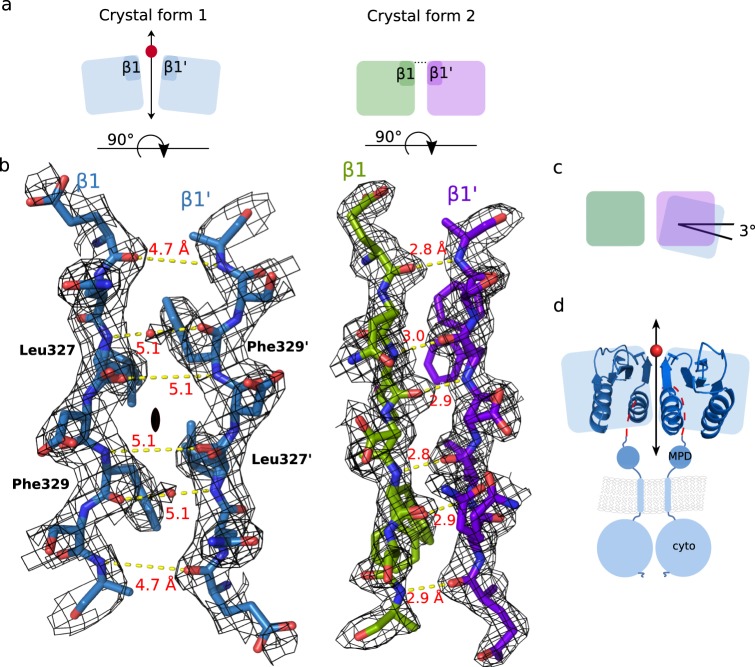
The GspL^fld^ dimer exhibits plasticity at the interface. (**a**) Schematic representation of the dimer of crystal form 1 viewed along the twofold axis (double headed arrow) and the dimer of crystal form 2 seen in the analogous way. Each block represents the whole ferredoxin-like domain and the depiction portrays this particular orientation of the dimer. The different proximity of interacting strands results in open (crystal form 1) and closed (crystal form 2) variant of the GspL^fld^ dimer. (**b**) The detailed view on the beta strands contributing to the interaction interfaces of crystal forms 1 and 2. The dramatically different inter-strand distances result in complementation of the interface with the extension of antiparallel β-sheet in case of crystal form 2 and lack of thereof in crystal form 1. The twofold axis is marked with a black diad symbol. The open variant of the crystal form 1 is mediated by coordination of a water molecule, indicated in dark red sphere. The two distinct scenarios can be fulfilled by the N-H of Leu327 and the C = O of Phe329′. The distances between main chains’ N and C = O groups are labeled with yellow dashed lines and reported in Å. The models are shown in the electron density 2Fo-Fc maps contoured at 2σ. (**c**) The plasticity of the GspL^fld^ reflected by interdomain tilting of 3°. (**d**) The dimer of crystal form 1 incorporated into the schematic representation of the GspL full-length dimer.

Nevertheless, the general conservation of the dimer interface does not guarantee retention of the same type of stabilizing interactions. Structural superpositions of GspL^fld^ as observed in crystal forms 1 and 2 and the dimer of *V*. *parahaemolyticus* reveal that the hallmark inter-domain β-sheet extension in *V*. *parahaemolyticus* GspL^fld^ and crystal form 2 is not observed in crystal from 1, despite fold conservation (Fig. 3 and Sup. Fig. S2b). The different inter-strand distances between β1 and β1′ in *P*. *aeruginosa* GspL^fld^ crystal forms result in two distinct conformations of the GspL^fld^ dimer (Fig. 4a). As shown in Fig. 4b, the distance between the main chain atoms of β1 and β1′ of crystal form 1 is in the range of 4.5–5.0 Å and is drastically greater than the equivalent interdomain in crystal form 2, which are instead within hydrogen bonding distances (2.8–3.0 Å). In this respect, the rather open dimer in crystal form 1 resembles the dimer conformation found in the asymmetric unit of EpsM^33^. The electron density of the inter-strand region of crystal form 1 has revealed the presence of an ordered water molecule positioned halfway between the main chain nitrogen of Leu327 and the carbonyl oxygen of Phe329′ (Fig. 4b, red sphere). The water molecule mediating this inter-strand distance sheds light on the lack of complementation of hydrophobic interface by extension of the β-sheet. It acts as both hydrogen bond donor and acceptor, to interact with the amide hydrogen of Leu327 and carbonyl oxygen of Phe329′, which are otherwise hydrogen bonded with each other in the closed form of GspL^fld^ (Fig. 4b, compare crystal form 1 and 2).

In this regard, crystal form 2 and the *V*. *parahaemolyticus* crystal form of GspL^fld^ appear as closed versions of the dimer whereas crystal form 1 exploits a novel conformation, resulting in an open variant of the interface. The opening is also reflected by the tilting of ferredoxin-like domain approximately by ~3°, with respect to the second monomer (Fig. 4c). In this way the crystal forms of GspL^fld^ presented here not only validate the dimeric interface, but also provide arguments for the potential plasticity of dimeric GspL participating in T2SS. Thus, the lack of stabilizing interactions such as hydrogen bonds and electrostatic interactions at the dimer interface, which are generally directional and bestow specificity, is most likely the reason for the observed conformational plasticity at the dimer interface.

### GspL^fld^ is a dimer in solution

To confirm the oligomeric state of GspL^fld^ in solution we exploited the proteolytic lability of GspL^peri^ in crystallization conditions to purify GspL^fld^ followed by analysis of GspL^fld^ by SEC-MALLS. In order to generate a relevant sample, purified GspL^peri^ was incubated in crystallization conditions over 48 hours and the N-terminus of the newly formed species was confirmed by Edman degradation analysis (Sup. Fig. S3a,b). Edman degradation analysis of the 10 kDa band (Sup. Fig. S3a) has revealed that the band consists of a mixture of two variants, starting at residues 286 and 295, and that the shorter species is the predominant one (Sup. Fig. S3b). In this regard, the analysis confirms that the newly generated protein fragment lacks the MPD (Fig. 1a) and therefore corresponds to GspL^fld^. We subsequently purified GspL^fld^ by SEC by following its elution by absorbance at 220 nm owing to its lack of aromatic aminio acids (Sup. Fig. S3c). Finally, purified GspL^fld^ was subjected to SEC-MALLS, which confirmed that GspL^fld^ is a dimer in solution (Sup. Fig. S3d).

### The N-terminus of GspL^peri^ is flexible

The presented distinct crystal structures of GspL^fld^ are lacking part of the MPD, as compared to the GspL^peri^ construct (Fig. 1b). None of the crystal lattices offers a rationale for the tetramerization propensity of GspL^peri^ in solution (Fig. 2b). In order to gain insights into the shape of the entire periplasmic part of GspL, but also to cross-validate the co-existence of tetramers and dimers in solution revealed by SEC-MALLS, small-angle X-ray scattering (SAXS) was employed.

**Figure 5:**
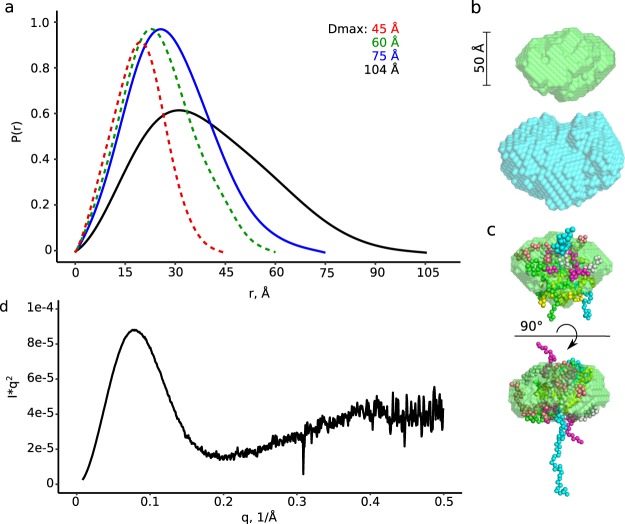
SEC-SAXS analysis validates the existence of dimers and tetramers of GspL^peri^ in solution. (**a**) The experimentally determined distance distribution functions of dimer and tetramer of GspL^peri^ (solid lines, blue and black) in comparison to the calculated P(r) functions of dimer and monomer (dashed lines, green and red) of crystal form 1. (**b**) *Ab initio* envelopes generated for dimer (green) and tetramer (cyan) (**c**) The dimeric envelope manually fitted with the dimer of crystal form 1 and an ensemble of most populous models of the N-terminus, presented as chains of spheres. (**d**) Kratky plot of the GspL^peri^ dimer.

As can be seen in Table 2, the molecular weights of the species from EEP and LEP (Fig. 1c) derived from our SEC-SAXS measurements agree with the masses for the tetrameric and dimeric molecular species obtained via SEC-MALLS experiments. The SAXS-derived molecular masses relate to the increasing size of the species, as reflected by the experimentally determined D_max_ (Fig. 5a, solid lines). As expected, these D_max_ values are greater than those calculated for the crystallographic dimer and monomer (Fig. 5a, dashed lines).

**Table 2:** SAXS data collection, analysis, and modelling.

		GspL^peri^	
		dimer	tetramer
	SASBDB code	SASDDB8	SASDDC8
**Data collection parameters**			
	Beamline	SWING, Soleil, Paris	
	Detector	PCCD170170 (AVIEX)	
	Beam geometry, mm^2^	1 × 0.5	
	Wavelength, Å	1.03	
	Mode	SEC	
	Column	4.6 × 300 mm Bio SEC-3	
	Initial concentration, mg/ml	13.7	9.0
	Injected volume, µl	50	
	Flow rate, ml/min	0.2	
	Temperature, °C	15	
**Software employed**			
	Primary data reduction	FOXTROT	
	Data processing	Primus, Gnom^55,56^	
	Ab initio analysis	DAMMIF^36^	
	validation and averaging	DAMAVER^57^	
	Modelling	EOM^38^	
	Computation of model intensities	Crysol^55^	
	3D graphics representation	Pymol	
**Structural parameters**			
**Guinier Analysis**			
	I(0)	0.037 ± 2.5e-5	0.039 ± 2.3e-5
	R_g_, Å	22.42 ± 0.03	31.75 ± 0.37
	Q_min_, 1/Å	0,0018	0,0025
	qR_g_max	1.23	1.23
	M from I(0), (ratio to predicted value)	1.30	1.05
**P(r) analysis**			
	I(0)	0.039 ± 0.91e-5	0.037 ± 0.27e-4
	R_g_, Å	22.43 ± 0.07	30.98 ± 0.03
	D_max_, Å	75	105
	q range, 1/Å	0.0018–0.517	0.0025–0.517
	M from I(0), (ratio to predicted value)	1.37	1.00
	Porod volume estimate, Å^3^ (ratio V/calc. MW)	30128 (1.08)	60526 (1.08)
	dry volume from sequence, Å^3^	33777	67554
**Molecular mass determination**			
	SAXS_MoW server, kDa	22.8	83
	Scatter software, kDa	32.7	63.9
	calculated from sequence, kDa	27.9	55.8
	determined by MALS, kDa	27.5	55.5
**Shape model-fitting results**			
**DAMMIF (default parameters, 20 models)**			
	q range for fitting, 1/Å	0.0018–0.517	0.0025–0.517
	Symmetry and anisotropy assumptions	P1, none	P1, none
	NSD (standard deviation), no. of clusters	1.036 (0.0022), 3	1.065 (0.0038), 4
	χ^2^ range	2.474–2.528	2.826–2.875
	Constant adjustment to intenities	Skipped, unable to detrmine	
	MW estimate (ratio to predicted value)	15.2 (0.54)	31.95 (0.57)
**EOM (default parameters, 10 000 models in initial ensemble, native-like models)**			
	χ^2^	5.788	NA
	number of representative structures	6	NA

Next, we used the SAXS data and the determined D_max_ values to calculate *ab initio* molecular envelopes for dimeric and tetrameric species using DAMMIF^36^. To generate the least biased envelopes possible, no symmetry was imposed. Nevertheless, both envelopes and in particular the corresponding to dimeric GspL^peri^ display features of an apparent 2-fold axis of symmetry (Fig. 5b,c). In addition, the averaged tetrameric envelope does appear to display symmetry higher than proper 2-fold symmetry. The volume of the dimeric envelope is substantially larger than the volume of the GspL^fld^ crystallographic dimer. In fact, the shape and the size of the envelope does not restrict the position of the folded domain. However, attempts to model the missing 4 kDa N-terminal fragment with CORAL^37^ were unsuccessful. We were compelled to consider that the N-terminus of GspL^peri^ might be flexible, a characteristic that is not readily clear from inspection of the Kratky plot (Fig. 5d), yet expected from the proteolytic susceptibility revealed in the crystallographic experiment. Therefore, the N-terminal 4 kDa of GspL^peri^ has been modeled as an ensemble of models via EOM^38^ (Fig. 5c). The ensemble is composed of six differently populated models, reflecting our inability to explain the envelope of the given size with two single polypeptide chains, 14 kDa each. Of note, the ensemble fits the independently generated envelope well, with accommodation of the dimeric crystal structure (Fig. 5c). Taken together, the much greater size of the *ab initio* dimeric envelope as compared to the volume of two folded 14 kDa chains, as well as proteolytic susceptibility of the membrane proximal domain indicate that the N-terminal part of GspL^peri^ is flexible in solution.

## Discussion

Three decades of studies on bacterial type II secretion systems have tremendously broadened our knowledge on bacterial secretion of folded proteins^15,39^, yet several outstanding questions have remained unanswered. One of the most urgent yet basic issues concerns the oligomeric state of the proteins composing functional T2SSs. Our solution studies complemented with two crystal structures of the periplasmic component of GspL provide insights into the oligomeric propensities and plasticity of this protein. Our in-solution studies have revealed that GspL^peri^ might exist in two oligomeric states: dimeric and tetrameric. Moreover, under the experimental conditions used the tetrameric state spontaneously converts into a dimeric form and the transition is concentration independent (Fig. 2). Yet, the tetramer has only been observed for the full-length periplasmic construct and crystal contacts in the three available crystal structures of GspL^fld^ do not hint at a higher-order oligomerization of the ferredoxin-like domain, consistent with the dimeric state of GspL^fld^ in solution (Fig. S3). Thus, our data point to the involvement of the membrane proximal domain of GspL in the tetramerization mechanism and implies superior stability of the dimer in solution.

GspL is a bitopic inner-membrane protein which interacts in the periplasm with at least one protein, GspM (Fig. 1a). Importantly, GspL and GspM share the same organization of periplasmic domains (Fig. 1a) and have been identified as structural homologues^21^. The MPD of GspL is predicted to possess two alpha helices with a weak coiled-coil tendency. The MPD of PilN, GspL^peri^ homologue from the type IV pilus system, has much more prominent coiled-coil region which has been suggested to drive the heterodimerization with PilO, GspM homologue^32^. The existence of PilN-PilO complex has been demonstrated both *in vitro* and *in vivo*^34,40^. Interestingly, no coiled-coil region is predicted for GspM. Also, while it is not entirely clear what might drive the GspM-GspL interaction, the involvement of both the periplasmic parts and transmembrane helices has been demonstrated^13^. All arguments considered, the coiled-coil region(s) of GspL might be exploited not for the interaction with GspM, but to drive tetramerization.

Analyzing the sequence of the predicted helix of MPD, which spans residues 281–296, one may recognize the heptad repeat motif starting at Leu282 (Fig. 1b). However, its positioning at the very beginning of the predicted helix might influence its stability. Importantly, in the course of crystallographic experiment the protease cleaved the chain within the motif meaning that the helix is not stable in the isolated periplasmic fragment. We hypothesize that the herein reported instability of the membrane proximal domain might underlie the transient character of the GspL^peri^ tetramer.

**Figure 6:**
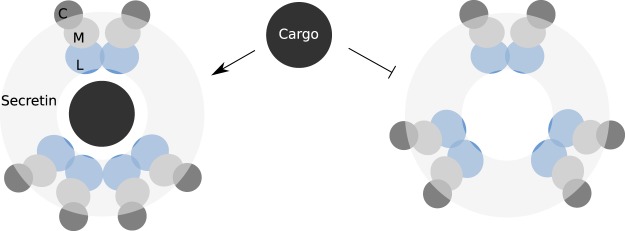
A model for substrate loading to the periplasmic vestibule of the type II secretion system. The drawing encompasses periplasmic domains of GspL (blue), GspM (grey), GspC (dark grey), the secretin channel (light grey) and a molecule of a cargo protein (black) viewed from the top of the secretion machinery. In the proposed model the transient tetramerization of GspL^peri^ is proposed as a mechanism to open the periplasmic vestibule and to enable entry of an exoprotein (left). In contrast, the channel is closed when the GspL^peri^ dimers are equivalently distributed (right).

In addition, the tendency of GspL^peri^ to tetramerize might reflect its functionality in the context of T2SS. We propose that it might act as a gate keeper at the periphery of the machinery. In the proposed model presented in Fig. 6, the even distribution of dimeric building blocks of GspL provides steric hindrance within the periplasmic vestibule, rendering it not permeable by folded proteins. In this regard, tetramerization could lead to transient opening of the channel to enable take up of a cargo protein.

In this work we presented two distinct crystal structures of GspL^fld^, which in combination with the crystal structure of GspL^fld^ from *Vibrio parahaemolyticus*^21^, provide direct evidence for the conservation of a hydrophobic dimer interface. The hydrophobic nature of the observed interactions renders the observed dimeric assembly as the minimal building block of GspL in an aqueous environment. At the same time, interrogation of the functional importance of such a highly hydrophobic interface by site-directed mutagenesis and in *in vivo* studies, presents with intrinsic challenges. This is because the disruption of extensive hydrophobic interfaces driving oligomerization would generally entail multiple mutations, typically in combinatorial fashion, in order to render the mutant protein expressible and soluble for biochemical and functional analysis. In fact, the absence of monomeric GspL in any of our preparations of GspL, even in very dilute solutions, is testament to the constitutive dimeric character of GspL. Therefore, GspL and all homologues thereof characterized to date, adopt a dimeric state as a minimum oligomeric state by all biochemical and biophysical standards. Notwithstanding complementation of the hydrophobic interface by extension of antiparallel β-sheet over two ferredoxin-like domains in cases of crystal form 2 and *V*. *parahaemolyticus* dimeric structure, crystal form 1 have adapted the open variant of dimer (Fig. 4). This unique structural feature creates a window of opportunity to discuss the plasticity of the GspL^fld^ dimeric interface.

The cytoplasmic domain of GspL interacts with the system ATPase, GspE^8,9^. The protein directly links system components localized in cytoplasm and periplasm^14^, and is considered to play the major role in conversion of chemical energy from ATP hydrolysis to mechanical energy utilized in secretion process^7^. The estimation of ΔG required for disruption of dimeric interface for all three crystallographic GspL^fld^ dimers, is in the range of 10–11 kcal/mol^41^. It is significantly more (by ~4 kcal) than energy originating from hydrolysis of a single molecule of ATP; however, this comparison is made to point out that GspL has a direct access to the source of energy. The indicated number reflects the cost of exposure of hydrophobic residues to water environment, what makes monomerization of GspL^fld^ an extremely unlikely scenario. Nevertheless, the GspL^fld^ dimer is capable of subtle opening of the interface, which does not affect its hydrophobic core but perturbs extension of antiparallel β-sheet, as documented by crystal form 1. We hypothesize that in the case of the T2SS *modus operandi* partial opening of the dimeric interface of GspL, much less energetically expensive than interface disruption, could be empowered by ATP hydrolysis. It might be followed by creation of another, slightly more or less hydrophobic interface, with a different interaction partner. Strikingly, the dimeric periplasmic interfaces of GspM and GspD share with GspL hydrophobic character^26,33^.

There always exists a risk of assigning biological relevance to crystal lattice contacts. However, we do not consider that the crystal form 1 open dimer is the result of crystal packing because the very same symmetry operation underlies the formation of the closed dimer of GspL^fld^ of *V*. *prahaemolyticus*^21^. Also, the lack of β-sheet extension is the most striking, yet not the only argument in favor of plasticity of the dimer. A more indirect hint came from the necessity to model the two dimer interface residues Leu327 and Phe329 into two conformations for each residue, in crystal forms 1 and 2, respectively. Furthermore, in *V*. *parahaemolyticus* GspL^fld^ dimer these positions are both occupied by aromatic residues which do not mutually interact via π-stacking but rather via van der Waals interactions, and which interact more with other interface residues than between each other. Altogether, the hydrophobic nature of the dimer interface dictates its mandatory character in the aqueous environment, yet a few structural features revealed by the models at atomic resolution offer strong evidence supporting molecular plasticity. In the context of the T2SS such a molecular property might be key for interactions between GspL and other T2SS proteins.

Importantly, the dimeric character of the GspL in combination with its peripheral positioning in the T2SS machinery enables us to address the overall stoichiometry of the T2SS. Viewed from the top, as in Fig. 6 model, the system possesses radial symmetry with a fold of a symmetry of the secretin channel. From our research it is evident that GspL might exist in the aqueous milieu in the form of dimer, or its multiplicity. The available data on interactions between the system components support 1:2 stoichiometry of the inner membrane platform and the secretin interaction, GspLMC:GspD^15^. In this way our results are in favor of the T2SS models with 12- or 16-fold symmetry of the secretin. The recent reports on secretins featuring not only 12- or 16- but also 14- and 15-fold symmetries^23–27^ are causing a reconsideration of our view of the overall arrangement of the T2SS. The dynamic character of the working machinery makes studies on this matter extremely challenging, therefore requiring integration and validation of the available data. We envisage that such approach will pave the way for a better understanding of the Gsp-dependent bacterial secretion.

## Methods

### Cloning and expression of recombinant GspL^peri^

The GspL^peri^ construct was created by amplification of residues 261–382 (numbering after uniprot database, GSPL_PSEAE entry P25060) by polymerase chain reaction (PCR) from genomic DNA of PAO1 *P*. *aeruginosa* strain kindly provided by prof. Jan Tommassen. The following primers were used, enabling introduction of Strep-tag at N-terminus:

Antisense: 5′-TCCTTGGATCCTTACTAACCTCCTATCACCAGGCGCGCGCTGAC-3′

The PCR product was ligated into pRSF 1b expression vector (Invitrogen) using NcoI and HindIII restriction sites.

For expression, *Escherichia coli* BL21 (DE3) (Invitrogen) cells were transformed with the DNA, and 25 ml of overnight preculture supplemented with kanamycin (50 μg/ml) was used to inoculate 1 l of Luria broth medium. The bacteria were grown until optical density of 0.6, measured at λ = 600 nm, at which they were induced with 0.1 mM isopropyl-β-D-1-galactosidase (IPTG), followed by 3 h long expression at 37 °C and harvesting by centrifugation. The collected cell pellet was stored in −80 °C.

### Protein purification

The frozen cell pellet was thawed and resuspended in a lysis buffer (50 mM TRIS, pH = 8.0 at 4 °C, 300 mM NaCl, hen egg white lysozyme: 1 mg/mg of pellet) supplemented with Complete (Roche) EDTA-free protease inhibitor cocktail. The cells were lysed by sonication followed by 30 min centrifugation at 75.000 g. The supernatant was decanted, filtered through a 0.22 μm syringe filter cap and loaded on Strep-tactin Superflow(R) matrix (IBA Lifesciences) preequilibrated with a wash buffer (WB; 50 mM TRIS, pH = 7.5 at 25 °C, 100 mM NaCl). The protein was washed with 15 column volumes (CV) of WB and eluted with 2xCV of WB containing 2.5 μM desthiobiotin (IBA Lifesciences). The eluate was concentrated and then subjected to size exclusion chromatography on Superdex 75 column (GE Healthcare), also preequilibrated with WB. The protein eluted in one or two monodisperse peaks (details in results section), which were collected and concentrated for further experiments. Sample purity was estimated to be >95%, based on SDS-PAGE analysis followed by Coomassie staining.

### SEC-MALLS measurements

The experiment was performed on a Wyatt Technology system, consisting of: HPLC unit with Superdex 200 increase column (GE Healthcare), UV detector (Shimadzu), a three-angle static light scattering detector (DAWN Theros) and refractometer (Optilab T-rEX). The pure concentrated sample was injected on the column preequilibrated with WB. Afterwards 200 μg bovine serum albumine in WB was run as a monodisperse reference protein. For molecular weight calculation a dn/dc value of 0.185 ml/g was used. Data analysis was performed with Astra V software.

### Crystallization, structure solution and refinement

The protein sample used for crystallization experiment originated from the LEP (see Fig. 1c). Crystallization trials were performed via vapor-diffusion method, in sitting drop geometry. The experiments were set up with the usage of Mosquito robot, by mixing 100 nl of the purified protein solution with a reservoir in 1:1 volume ratio. The crystallization drops were equilibrated against 40 μl reservoir solutions and incubated at 20 °C. For broad screening of reservoir compositions commercial sparse matrices (Molecular Dimensions, Hampton Research) were used. The initial concentration used in the screens was 35 mg/ml and was determined with the aid of Pre-Crystallization Test (Hampton Research).

The initial crystalline hits appeared after one- and three-week-long incubation in conditions containing: (1) 0.2 M L-Proline, 0.1 M HEPES, pH = 7.5, 10% w/v PEG 3350 and (2) 0.3 M KBr or 0.2 M MgCl_2_, 0.1 M sodium cacodylate, pH = 6.5, 8% w/v γ-PGA. The final datasets which served for structure solution were collected from single crystals grown in optimized conditions: (1) 0.2 M L-Proline, 0.1 M HEPES, pH = 7.5, 8% w/v PEG 3350, protein sample at 34 mg/ml; grown after a week (2) 0.36 M KBr, 0.1 M sodium cacodylate, pH = 6.5, 4% w/v α-PGA, protein sample at 36 mg/ml protein concentration; grown after 3 months. As the protein crystallized in two different crystal forms, the structures determined from the crystals grown in the above-mentioned conditions and the corresponding datasets, are referred to as crystal form 1 (I-centered tetragonal Bravais lattice) and crystal form 2 (primitive orthorhombic Bravais lattice).

In order to determine identity of the crystallized species multiple crystals were grown in the PEG-containing condition. After growth over a week the crystals were harvested, washed twice in cryoprotectant solution, dissolved in purification buffer and run on Tris/Tricine precast gel (Biorad). The staining of the gel was handled with Pierce Silver Stain kit (Thermo). Sizes of the construct were estimated in the reference to molecular weight marker (Biorad).

Prior to data collection crystal form 2 crystals were transferred through cryoprotectant solution containing mother liquor with 14% (w/v) PEG 3350 and 20% (v/v) glycerol, while crystal form 1 crystals were supplemented in their native drop with ethylene glycol, to the final 18% (v/v) concentration.

Data collections under cryogenic conditions were performed at Proxima2 microfocus beamline, Soleil Synchrotron. The datasets were processed in XDS^42^. The structures were solved via maximum-likelihood molecular replacement (MR) protocols in Phaser as implemented in Phenix^43,44^. An initial MR solution for crystal form 2 was found using a model based on the structure of GspL from *Vibrio parahaemolyticus* (PDB code: 2w7v), identified with HHPred^45^ and truncated to common Cβ atoms using Phenix Sculptor^46^. The partially refined solution of crystal form 2 served later on for determining the structure of crystal form 1. The models were built manually in multiple building rounds in Coot^47^, alternating with refinement carried out with Phenix.Refine^48^ and validated in Coot and MolProbity^49^.

Data collection and refinement statistics are listed in Table 1. The dataset for crystal form 2 was diagnosed with translational non-crystallographic symmetry (tNCS), which often hinders crystallographic refinement resulting in higher than normal crystallographic R-factors^50–53^.

### Edman degradation

The purified protein was concentrated to 5.2 mg/ml and stored at 20 °C for a week. Afterwards it was run on Tris/Tricine gel, blotted on PVDF membrane (Applied Biosystems) and the membrane was stained with Coomassie R-250. Edman degradation was performed on a 10 kDa degradation band, sequentially washed with water and methanol. N-terminal sequence determination was performed by automated Edman degradation on a Procise model 494 sequencer instrument, equipped with an on-line HPLC system consisting of a 140 C Microgradient pump and a 785 A programmable absorbance detector (all from Applied Biosystems). The analysis was performed with acid delivery in the gas phase on the protein sample.

### SEC-SAXS measurement and primary analysis

GspL^peri^ eluting in early and late elution peaks was collected, concentrated to 9.0 and 13.7 mg/ml, and flash frozen. The samples were thawed shortly prior to SEC-SAXS data collection performed at SWING beamline, Soleil Synchrotron. The measurements were carried out at 283 K, with the employment of 4.6 × 300 mm Bio SEC-3 column with 300 Å pore size (Agilent) and the scattering signal was collected within a momentum transfer range s 0.01 Å > s > 0.6 Å. In order to be used for modeling, the data were buffer subtracted and reduced in Foxtrot software, developed at Soleil Synchrotron. 12–15 curves were chosen for averaging, based on stability of radius of gyration (R_g_) throughout the elution peak. Afterwards the SAXS data were processed with the ATSAS package^54^. The extrapolation to infinite dilution and the following R_g_ and I_0_ determination from Guinier region were performed in PRIMUS^55^, and in parallel, for real-space transformed data, in GNOM^56^. In addition, the distance distribution function P(r) calculated in GNOM served to determine the maximum particle dimension D_max_.

### SAXS modelling

The calculated *P(r)* function was used as input for *ab initio* modeling with DAMMIF from ATSAS suite^36,57^. The envelopes were modeled with no symmetry assumed. Afterwards, EOM^38^ was implemented to model the N-termini of the dimer, using the scattering data within the range 0.01 Å > s > 0.5 Å, and the dimer of crystal form 1 as a rigid body. The defined core symmetry was P2 and originated from the symmetry of the used rigid body. The overall symmetry was not restricted but allowed (option: mix, fraction: 0.5). Finally, the dimeric ensemble generated with EOM was manually superimposed on its DAMMIF generated envelope in PyMOL (The PyMOL Molecular Graphics System, Version 2.0 Schrödinger, LLC).

## Electronic supplementary material

Supplementary Figures

## Data Availability

The atomic coordinates and structure factors (codes 5n7l and 6ghu) have been deposited in the Protein Data Bank (http://www.rcsb.org/) and the SAXS curves have been deposited in the Small-Angle Scattering Biological Data Bank (http://www.sasbdb.org/) with accession codes SASDDB8 (dimer) and SASDDC8 (tetramer).

## References

[1] Costa TRD (2015). Secretion systems in Gram-negative bacteria: structural and mechanistic insights. Nat. Rev. Microbiol..

[2] Thomassin J, Moreno JS, Guilvout I, Nhieu GT (2017). Van. The trans-envelope architecture and function of the type 2 secretion system: new insights raising new questions. Mol. Microbiol..

[3] Kung VL, Ozer EA, Hauser AR (2010). The accessory genome of Pseudomonas aeruginosa. Microbiol. Mol. Biol. Rev..

[4] WHO. Global priority list of antibiotic-resistant bacteria to guide research, discovery, and development of new antibiotics. http://www.who.int/medicines/publications/WHO-PPL-Short_Summary_25Feb-ET_NM_WHO.pdf?ua=1 (2017).

[5] Bleves S (2010). Protein secretion systems in Pseudomonas aeruginosa: A wealth of pathogenic weapons. Int. J. Med. Microbiol..

[6] Filloux A (2011). Protein Secretion Systems in Pseudomonas aeruginosa: An Essay on Diversity, Evolution, and Function. Front. Microbiol..

[7] Korotkov KV, Sandkvist M, Hol WGJ (2012). The type II secretion system: biogenesis, molecular architecture and mechanism. Nat. Rev. Microbiol..

[8] Sandkvist M, Bagdasarian M, Howard SP, DiRita VJ (1995). Interaction between the autokinase EpsE and EpsL in the cytoplasmic membrane is required for extracellular secretion in Vibrio cholerae. EMBO J..

[9] Ball G (1999). Assembly of XcpR in the Cytoplasmic Membrane Is Required for Extracellular Protein Secretion in Pseudomonas aeruginosa. J. Bacteriol..

[10] Robert V, Filloux A, Michel GPF (2005). Subcomplexes from the Xcp secretion system of Pseudomonas aeruginosa. FEMS Microbiol. Lett..

[11] Michel G, Bleves S, Ball G, Lazdunski A, Filloux A (1998). Mutual stabilization of the XcpZ and XcpY components of the secretory apparatus in Pseudomonas aeruginosa. Microbiology.

[12] Robert V, Hayes F, Lazdunski A, Michel GPF (2002). Identification of XcpZ Domains Required for Assembly of the Secreton of Pseudomonas aeruginosa. J. Bacteriol..

[13] Lallemand M (2013). Dynamic Interplay between the Periplasmic and Transmembrane Domains of GspL and GspM in the Type II Secretion System. PLoS One.

[14] Gray MD, Bagdasarian M, Hol WGJ, Sandkvist M (2011). *In vivo* cross-linking of EpsG to EpsL suggests a role for EpsL as an ATPase-pseudopilin coupling protein in the Type II secretion system of Vibrio cholerae. Mol. Microbiol..

[15] McLaughlin LS, Haft RJF, Forest KT (2012). Structural insights into the Type II secretion nanomachine. Curr. Opin. Struct. Biol..

[16] Nivaskumar M, Francetic O (2014). Type II secretion system: A magic beanstalk or a protein escalator. Biochim. Biophys. Acta.

[17] Chang Y-W (2016). Architecture of the type IVa pilus machine. Science (80-.)..

[18] Sampaleanu LM (2009). Periplasmic domains of Pseudomonas aeruginosa PilN and PilO form a stable heterodimeric complex. J. Mol. Biol..

[19] Leighton TL, Dayalani N, Sampaleanu LM, Howell PL, Burrows LL (2015). Novel Role for PilNO in Type IV Pilus Retraction Revealed by Alignment Subcomplex Mutations. J. Bacteriol..

[20] Abendroth J, Bagdasarian M, Sandkvist M, Hol WGJ (2004). The structure of the cytoplasmic domain of EpsL, an inner membrane component of the type II secretion system of Vibrio cholerae: an unusual member of the actin-like ATPase superfamily. J. Mol. Biol..

[21] Abendroth J, Kreger AC, Hol WGJ (2009). The dimer formed by the periplasmic domain of EpsL from the Type 2 Secretion System of Vibrio parahaemolyticus. J. Struct. Biol..

[22] Abendroth J, Murphy P, Sandkvist M, Bagdasarian M, Hol WGJ (2005). The X-ray structure of the type II secretion system complex formed by the N-terminal domain of EpsE and the cytoplasmic domain of EpsL of Vibrio cholerae. J. Mol. Biol..

[23] Koo J, Lamers RP, Rubinstein JL, Burrows LL, Howell PL (2016). Structure of the Pseudomonas aeruginosa Type IVa Pilus Secretin at 7.4 Å. Structure.

[24] Yan Z, Yin M, Xu D, Zhu Y, Li X (2017). Structural insights into the secretin translocation channel in the type II secretion system. Nat. Struct. Mol. Biol..

[25] Hay ID, Belousoff MJ, Lithgow T (2017). Structural Basis of Type 2 Secretion System Engagement between the Inner and Outer Bacterial Membranes. MBio.

[26] Van Der Meeren R (2013). New Insights into the Assembly of Bacterial Secretins. Structural Studies of the Periplasmic Domain of XcpQ from Pseudomonas aeruginosa. J. Biol. Chem..

[27] Reichow SL, Korotkov KV, Hol WGJ, Gonen T (2010). Structure of the cholera toxin secretion channel in its closed state. Nat. Struct. Mol. Biol..

[28] Campos M, Cisneros Da, Nivaskumar M, Francetic O (2013). The type II secretion system - a dynamic fiber assembly nanomachine. Res. Microbiol..

[29] Lu C, Korotkov KV, Hol WGJ (2014). Crystal structure of the full-length ATPase GspE from the Vibrio vulnificus type II secretion system in complex with the cytoplasmic domain of GspL. J. Struct. Biol..

[30] Gu S, Shevchik VE, Shaw R, Pickersgill RW, Garnett JA (2017). The role of intrinsic disorder and dynamics in the assembly and function of the type II secretion system. Biochim. Biophys. Acta - Proteins Proteomics.

[31] Felix J (2013). Human IL-34 and CSF-1 establish structurally similar extracellular assemblies with their common hematopoietic receptor. Structure.

[32] Felix J (2016). Structural basis of GM-CSF and IL-2 sequestration by the viral decoy receptor GIF. Nat. Commun..

[33] Abendroth J, Rice AE, McLuskey K, Bagdasarian M, Hol WGJ (2004). The crystal structure of the periplasmic domain of the type II secretion system protein EpsM from Vibrio cholerae: the simplest version of the ferredoxin fold. J. Mol. Biol..

[34] Karuppiah V, Collins RF, Thistlethwaite A, Gao Y (2013). & Derrick, J. P. Structure and assembly of an inner membrane platform for initiation of type IV pilus biogenesis. Proc. Natl. Acad. Sci..

[35] Luo, J., Liu, Z., Guo, Y. & Li, M. A structural dissection of large protein-protein crystal packing contacts. *Sci*. *Rep*. **5**, 10.1038/srep14214 (2015). 10.1038/srep14214 PMC4572935 26370141

[36] Franke D, Svergun DI (2009). DAMMIF, a program for rapid ab-initio shape determination in small-angle scattering. J. Appl. Crystallogr..

[37] Petoukhov MV (2012). New developments in the ATSAS program package for small-angle scattering data analysis. J. Appl. Crystallogr..

[38] Bernado P, Mylonas E, Petoukhov MV, Blackledge M, Svergun DI (2007). Structural characterization of flexible proteins using small-angle X-ray scattering. J. Am. Chem. Soc..

[39] Tommassen J (1992). Protein secretion in Pseudomonas aeruginosa. FEMS Microbiol. Lett..

[40] Leighton TL, Yong DH, Howell PL, Burrows LL (2016). Type IV Pilus Alignment Subcomplex Proteins PilN and PilO Form Homo- and Heterodimers *in Vivo*. J. Biol. Chem..

[41] Krissinel E, Henrick K (2007). Inference of Macromolecular Assemblies from Crystalline State. J. Mol. Biol..

[42] Kabsch WXDS (2010). Acta Crystallogr. Sect. D Biol. Crystallogr..

[43] McCoy AJ (2007). Phaser crystallographic software. J. Appl. Crystallogr..

[44] Adams PD (2010). PHENIX: A comprehensive Python-based system for macromolecular structure solution. Acta Crystallogr. Sect. D Biol. Crystallogr..

[45] Biegert A, Lupas AN (2005). The HHpred interactive server for protein homology detection and structure prediction. Nucleic Acids Res..

[46] Bunkóczi G, Read RJ (2011). Improvement of molecular-replacement models with Sculptor. Acta Crystallogr. Sect. D.

[47] Emsley P, Lohkamp B, Scott WG, Cowtan K (2010). Features and development of Coot. Acta Crystallogr. Sect. D Biol. Crystallogr..

[48] Afonine PV, Ralf W, Headd JJ, Thomas C (2012). Towards automated crystallographic structure refinement with phenix.refine. Acta Crystallogr. Sect. D.

[49] Chen VB (2010). MolProbity: All-atom structure validation for macromolecular crystallography. Acta Crystallogr. Sect. D Biol. Crystallogr..

[50] Chook YM, Lipscomb WN, Hengming KE (1998). Detection and use of pseudo-translation in determination of protein structures. Acta Crystallogr. Sect. D Biol. Crystallogr..

[51] Oksanen E (2006). Reindeer b-lactoglobulin crystal structure with pseudo-body-centred noncrystallographic symmetry. Acta Crystallogr. Sect. D Biol. Crystallogr..

[52] Read RJ, Adams PD, McCoy AJ (2013). Intensity statistics in the presence of translational noncrystallographic symmetry. Acta Crystallogr. Sect. D Biol. Crystallogr..

[53] Sundlov JA, Gulick AM (2013). Structure determination of the functional domain interaction of a chimeric nonribosomal peptide synthetase from a challenging crystal with noncrystallographic translational symmetry. Acta Crystallogr. Sect. D Biol. Crystallogr..

[54] Franke D (2017). ATSAS 2.8: A comprehensive data analysis suite for small-angle scattering from macromolecular solutions. J. Appl. Crystallogr..

[55] Konarev PV, Volkov VV, Sokolova AV, Koch MHJ, Svergun DI (2003). *PRIMUS*: a Windows PC-based system for small-angle scattering data analysis. J. Appl. Crystallogr..

[56] Svergun DI (1992). Determination of the Regularization Parameter in Indirect- Transform Methods Using Perceptual Criteria. J. Appl. Crystallogr..

[57] Volkov VV, Svergun DI (2003). Uniqueness of ab initio shape determination in small-angle scattering. J. Appl. Crystallogr..

