# Supplementary material for: Structure and oligomerization of the periplasmic domain of GspL from the type II secretion system of Pseudomonas aeruginosa
Source: Sci Rep. 2018 Nov 13;8:16760. doi: 10.1038/s41598-018-34956-w (PMC6233222; doi:10.1038/s41598-018-34956-w)
Supplement: Supplementary file 1 — Supplementary Figures [file 41598_2018_34956_MOESM1_ESM.pdf]

Supplementary information

Structure and oligomerization of the periplasmic domain of GspL from the type II secretion system of *Pseudomonas aeruginosa*

Aleksandra Fulara[1,2], Isabel Vandenberghe[3], Randy J. Read[4], Bart Devreese[3], Savvas N. Savvides*[1,2]

Affiliations:
[1]Unit for Structural Biology, Department of Biochemistry and Microbiology, Ghent University, 9052 Ghent (Zwijnaarde), Belgium.
[2]VIB-UGent Center for Inflammation Research, 9052 Ghent (Zwijnaarde), Belgium.
[3]Laboratory for Microbiology, Department of Biochemistry and Microbiology, Ghent University, 9000 Ghent, Belgium.
[4]Department of Haematology, Cambridge Institute for Medical Research, University of Cambridge, Cambridge CB2 0XY, United Kingdom.
*Correspondence: savvas.savvides@ugent.be

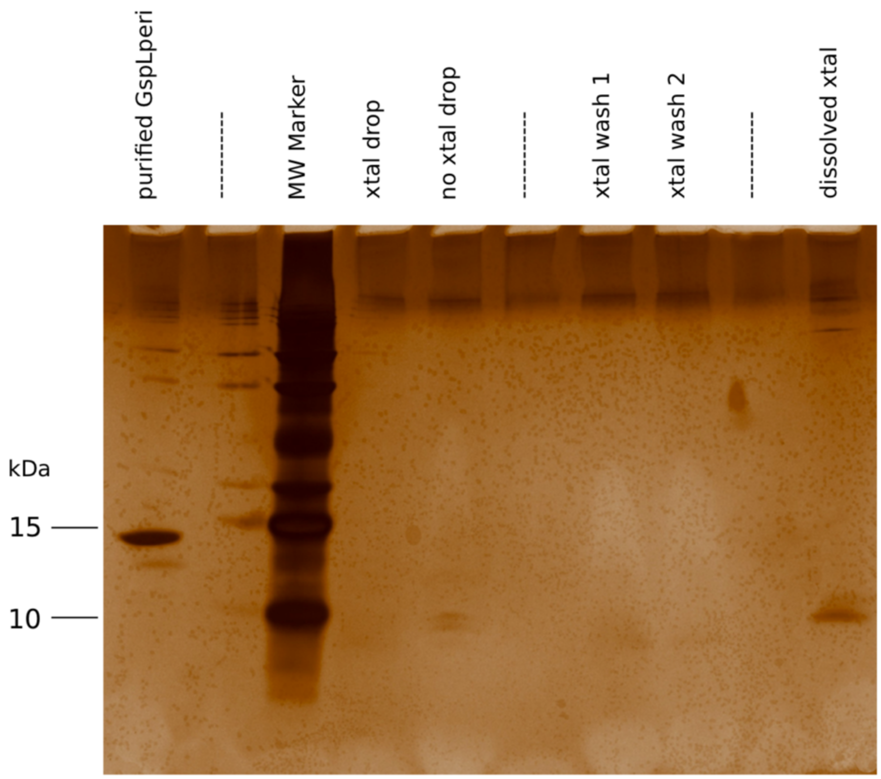

Figure S1. The N-terminus of GspL^{peri} is proteolytically cleaved in the course of crystallization. Silver-stained SDS-PAGE reveals significant mass difference between the protein material used for crystallization (purified GspL^{peri}) and protein crystal content (dissolved crystal). Presence of the same low MW protein band in a drop in which crystallization did not take place confirms that the crystal had grown from the proteolytically processed protein sample.

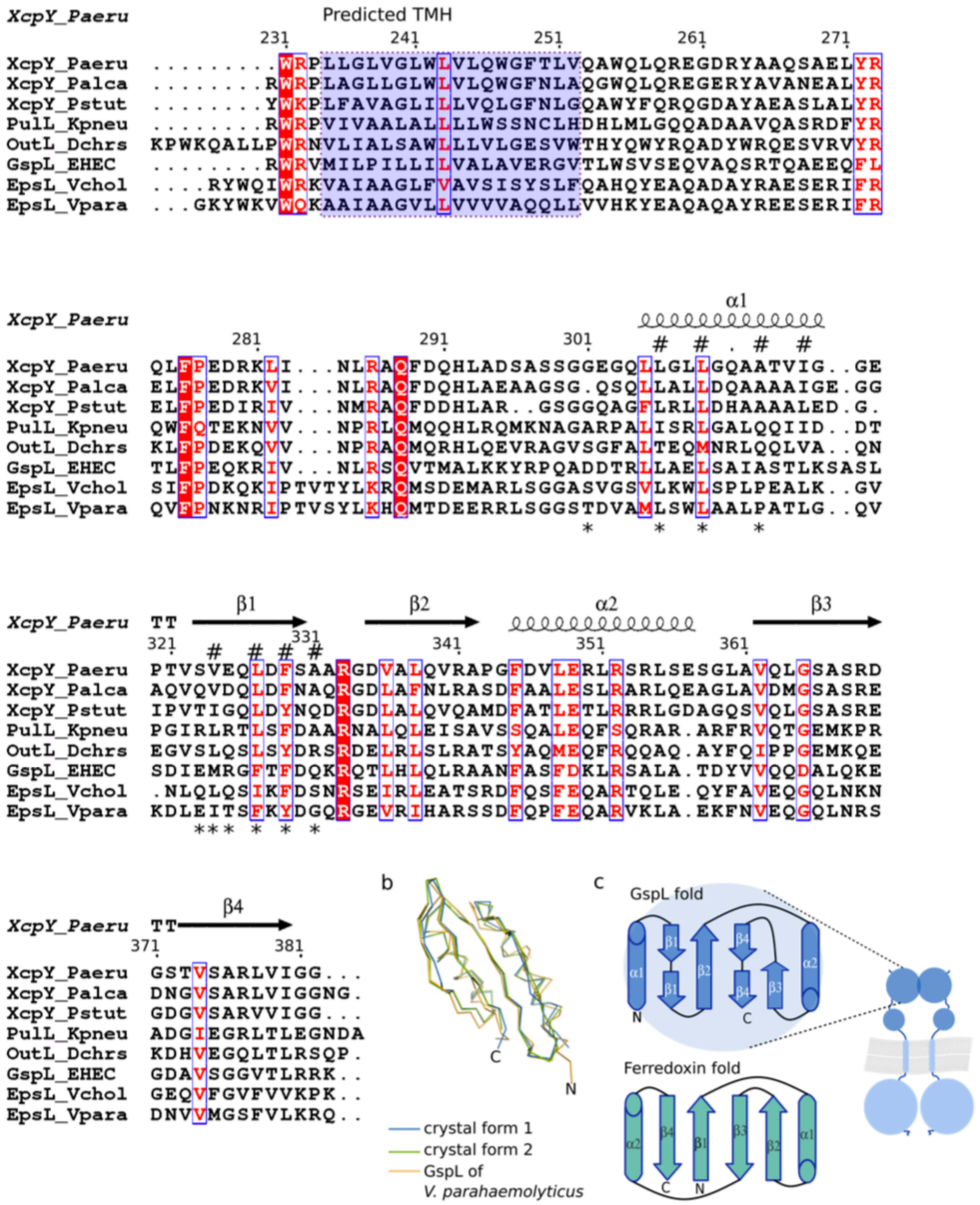

Figure S2. Structure-based sequence alignment of GspL sequences and structures from different species of Gram-negative bacteria. (a) The sequences are annotated with the secondary structure elements of GspL^{fld} (XcpY) of *P. aeruginosa*. Species-specific names are used for GspL from *Pseudomonas aeruginosa* (XcpY), *Pseudomonas alcaligenes* (XcpY), *Pseudomonas stutzeri* (XcpY), *Klebsiella pneumoniae* (PulL), *Dickeya chrysanthemi* (OutL), enterohemorrhagic *Escherichia coli* (GspL), *Vibrio cholerae* (EpsL) and *Vibrio parahaemolyticus* (EpsL). Residues participating to the dimeric interface in GspL^{fld} crystal structures presented in this manuscript are marked with #, whereas residues contributing to the interface of GspL of *V. parahaemolyticus* are marked with *. Figure made using ESPript 3.0. (b) Structural superposition of GspL^{fld} monomeric subunits from all available crystal structures, as presented in Figure 3, with respect to the monomer of crystal form 1 of GspL^{fld}. The root -mean-square deviation (RMSD) of the Cα atomic coordinates (chain A) of crystal form 2 and *V. parahaemolyticus* (60) is 0.42 Å (over 61 Cα atoms) and 1.25 Å (over 60 Cα atoms), with respect to crystal form 1. (c) Topology diagram of the ferredoxin fold and its permutation exploited by the periplasmic domain of GspL.

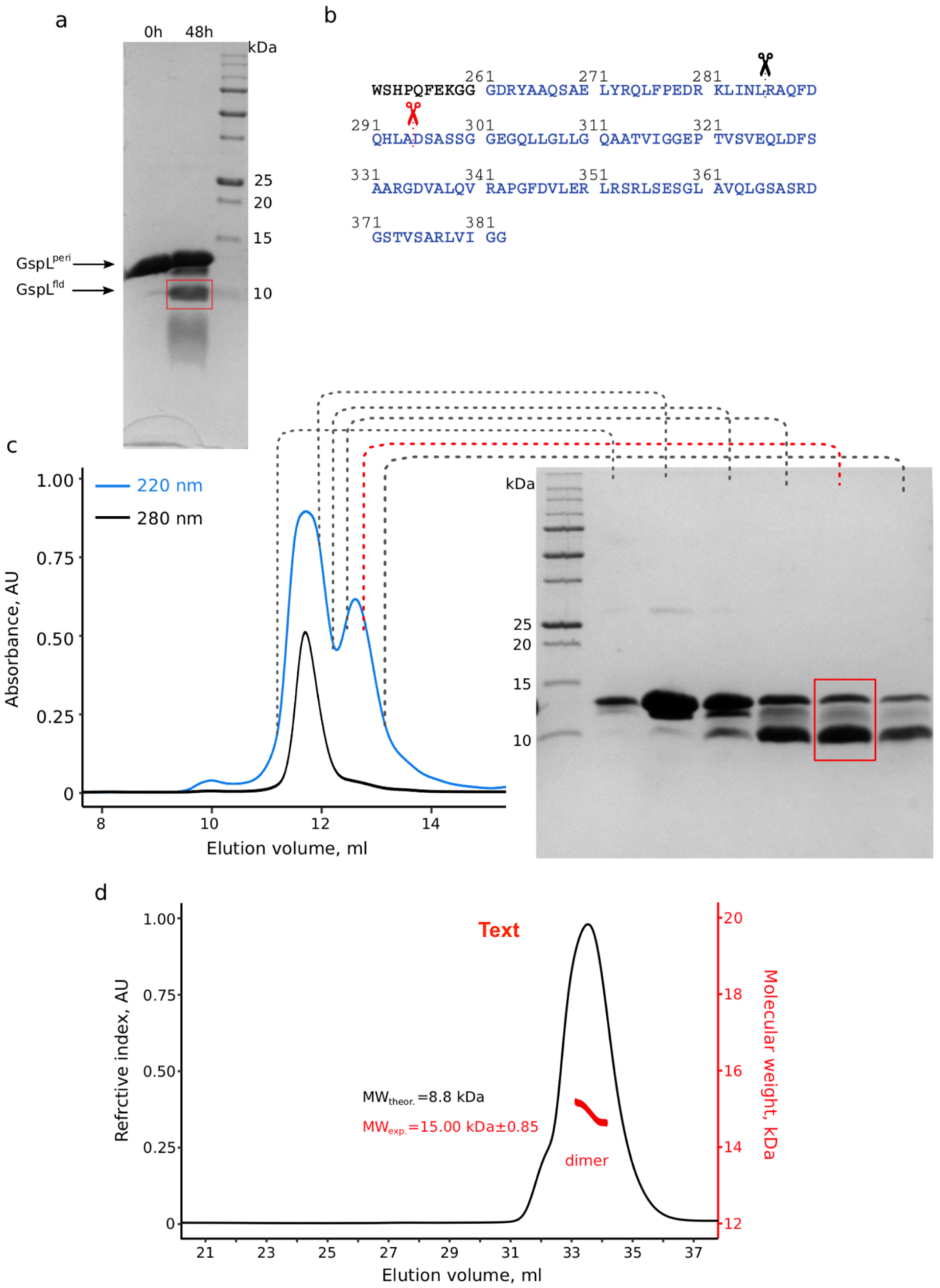

Figure S3. Gsp^{fld} is a dimer in solution. (a) Proteolytic cleavage of GspL^{peri} after incubation for 48 hours in crystallization condition. (b) Sequences of proteolytic species constituting the 10 kDa band, marked as GspL^{fld} in a, in reference to the sequence of the GspL^{peri} construct. The N-termini of the two identified chains are indicated with scissors. The red scissors mark the dominant species. (c) Size-exclusion chromatogram of the sample after 48 hours and corresponding SDS-PAGE analysis. The fraction marked in red was further propagated in SEC-MALLS experiment. (d) Molecular weight determination of GspL^{fld} by SEC-MALLS. The theoretical MW of the dominant species calculated from the sequence is 8.8 kDa.
